# Job Insecurity, Family Functionality and Mental Health: A Comparative Study between Male and Female Hospitality Workers

**DOI:** 10.3390/bs10100146

**Published:** 2020-09-24

**Authors:** Esperanza Vargas-Jiménez, Remberto Castro-Castañeda, Esteban Agulló Tomás, Raúl Medina Centeno

**Affiliations:** 1 Department of Psychology, Coast University Center, University of Guadalajara, Puerto Vallarta, Jalisco 48280, Mexico; rembert@cuc.udg.mx; 2 Department of Psychology, Universidad de Oviedo, 33003 Oviedo, Spain; estomas@uniovi.es; 3 Department of Communication and Psychology, Ciénega University Center, University of Guadalajara, Ocotlán, Jalisco 1115, Mexico; topraul2002@gmail.com

**Keywords:** job insecurity, precariousness, labor gap, hospitality industries, mental health, family functionality

## Abstract

The aim of the present article is to compare the family functionality, mental health and job insecurity of employees of the hospitality industry in Puerto Vallarta and Bahía de Banderas, in México. This is a quantitative and cross-sectional study. The sample was selected by non-probabilistic sampling for convenience and comprised a total of 914 people, of whom 438 were women (47.92%) and 476 were men (52.08%). The women surveyed reported more somatic symptoms, anxiety, insomnia and social dysfunction compared to men, which allows the conclusion that their mental health is vulnerable; meanwhile, men showed better perception of family functionality, a positive factor that reveals the family as a potential support factor that reduces stress, anxiety and improves men’s mental health. Another result reveals that the gender structure permeates the hotel sector, inequalities in the type of contract and income are corroborated, and the existence of a sexual division of labor to the detriment of women is confirmed, as they are mostly employed in low-skilled jobs that reproduce domestic tasks, particularly those related to cleaning and food service tasks.

## 1. Introduction

A job represents more than just a means for obtaining the basic sustenance that every human being needs to survive—it is also a way of creating a sense of belonging and is a fundamental part of the healthy psychosocial development of an individual. However, for a job to fulfill these functions and stimulate a person’s development and the development of a society, it is essential that it meets a series of basic criteria; it has to be a fair job [1,2].

Juan Somavia, the ex-director of the International Labor Organization (ILO), first introduced the concept of fair employment in 1999. He proposed four objectives on which we should concentrate in order to obtain fair employment: labor rights, job opportunities, social security and social dialogue. Each one of the proposed objectives looks to fulfill other goals related mainly to social inclusion, poverty eradication, democracy strengthening, integral development and personal realization [3].

The figure of precarious employment stems from this debate. Contrary to fair employment, precarious employment is characterized by being short lived, with a high risk of unemployment, scarce or inexistent capacity of the employee to control his or her employment conditions and a lack of social benefits. In consequence, employees are vulnerable to different forms of discrimination, they have inadequate labor conditions and they are badly paid [4,5,6,7].

One of the consequences of employment precariousness is a phenomenon known as job insecurity, defined as the subjectively experienced anticipation of a fundamental and involuntary event related to job loss [8]. Shoss [9] updates the conceptualization of this phenomenon as it is perceived today and adds that job insecurity is a perceived threat to the continuity and stability of employment.

Following these ideas, De Witte and Cuyper [10] add that job insecurity is accompanied by a lack of certainty about the future. This is because employment instability impedes the realization of personal and/or family plans due to the uncertainty regarding whether the necessary financial stability will be available in the future in order to achieve goals that have been established. Because it is a personal perception, each worker will analyze and perceive an employment situation differently. Even in identical circumstances, two people can have radically different points of view on their job. This variability will be a function of different personal, family and social factors such as personality, sex, age, position in the job market, family situation and country economics, among others.

Regarding the negative effects that those who live with job insecurity experience, a relationship between physical and mental health, general wellness and employment conditions has been observed [8,11,12,13]. This means that poorer employment conditions will result in poorer health. There is also evidence that, in many cases, work does not guarantee the fulfillment of basic necessities and that many workers are at risk of being catalogued as excluded socially. This is confirmed by an increase in the phenomenon of poor workers. For example, data from 2017 indicate that eight out of 10 young adults employed in Mexico do not have enough income to acquire basic nutrition expenses [14], placing them at the poverty threshold. Job insecurity also depletes confidence in a business’ management and its employees’ commitment to it. This is related to an important reduction in performance and productive behavior and also in a documented increase in employee desire to resign [1,6,15,16].

In relation to this, the job market confers especially precarious conditions upon women, putting them at greater risk of suffering from job insecurity. This has been noted by some international organizations. For example, the Organization for Economic Co-Operation and Development (OECD) [17] has reported on the inequalities in employment and rescaled the importance of studying and improving labor conditions for women because this group is subject to a gap in the field of employment that places them under vulnerable conditions.

In line with this, the OECD [17] points out that integral nation development will not be achieved if topics on gender equality are not included in public politics and governmental agenda. It states that that the pursuit of gender equality should be a priority in order to attain sustainable and inclusive growth that would benefit all citizens (Section 4). Thus, studies that aim to understand and address women’s necessity to improve quality of life should be a priority.

Despite women’s participation in the job market being closer to that of men in the last few decades, some institutions, like the National Institute of Statistics and Geography of Mexico (Instituto Nacional de Estadística y Geografía in Spanish, known as INEGI) and the OECD, agree that there is still a long road ahead before labor gender equality is obtained. For example, they state that, in a variety of countries, women have fewer opportunities than men to have a paid job. This is because gender stereotypes continue to establish that women should be committed to their own household without pay. In relation to this, women that do incorporate themselves into the workforce are subject to different forms of discrimination and receive a smaller salary than men [9,18].

Regarding salary, women who work earn on average less than men that have the same academic level and occupy the same position at work. This phenomenon is known as the wage gap. Statistics for the OECD indicate that, in countries that are members of the OECD, women earn on average 15% less than men [17]. This practice is considered a form of job discrimination and puts women at a disadvantage against male colleagues.

With respect to the types of jobs that women obtain, the OECD [17] indicates that women have a smaller chance to be business leaders and that businesses owned by women tend to earn less than those that are owned by men. In other words, women are typically employees and even self-employed women encounter greater obstacles than self-employed men.

To understand this information, the OECD offers 2016 data stating that, in Mexico, 34.3% of women work as managers. This allows us to have an idea of what kind of employment women have access to. In relation to wage inequality between men and women, the ILO [19] states that, worldwide, women only earn 0.77 USD for each dollar earned by a man and that, if this continues, it will take the world at least 70 years to close the wage gap.

Following these ideas, the National Council of Evaluation of Development Politics (in Spanish, Consejo Nacional de Evaluación de la Política de Desarrollo Social, CONEVAL) [20] indicates that, in 2016, Mexican women earned one fifth less than men, even with the same educational level. It also states that women on the job market still suffer from inequities compared to the employment conditions of men and that, in 2016, for every 100 employed men that had social security benefits, only 62 employed women were in the same situation. This gap is emphasized among the population in conditions of poverty, in which, for every 100 working men with social security, only 49 women have access to these employee benefits.

In this direction, according to the United Nations [21], women receive on average 24% less salary than men. In relation to this, regarding countries that register data on unemployment rates by education level, 85% of the 92 countries agree that women with a high education level have greater unemployment levels than men with the same education level.

Findings on gender gaps indicate that paternity and maternity play a crucial role in gender equality. Specifically, maternity has greater negative effects than paternity on participation in the job market, in wages and in professional progress [17]. It has been determined that there are barriers that impede women’s advancement into positions of power, decision making and visibility. Thus, a variety of metaphors have emerged to describe this phenomenon, such as the glass ceiling, scissor effect or sticky floors. These analogies describe how the job market for women is still restricted to lower qualification positions [22].

To understand the differences in employment trajectories between men and women, we refer to Pierre Bourdieu [23], who states that sexual division of work is cemented in the patriarchy, which he catalogues as a social construct that implies that women remain in a state of feminine oppression. This oppression is accomplished in diverse manners and is reproduced through perception schemes that are internalized in men and in women. Bourdieu [23] also adds that sexual division of labor assigns each sex a different way of relating, thus creating differences that go beyond anatomical and physiological conditions. These types of conducts are known as gender roles and they impact the lives of human beings because they predetermine preferences, conceptions and ideologies in both sexes. It is important to note that this principal is conceived, reaffirmed and perpetuated within the family and also through institutions like the state, the church and school.

The ideology that supports the patriarchy explains why many women are relegated to positions that consist of care for others (children, the elderly, the sick or the disabled) or consist of activities that resemble household activities. Women are thought to perform roles that do not consist of powerful positions or of decision-making. This is the result of a phenomenon known as the double working day (or double presence). The double working day has its foundation in traditional gender rules, in heteronormativity and marriage and has the objective of procreation, From these positions, a domestic charge is assumed and child care is the responsibility only of women because they are physically able to gestate and give birth. Nevertheless, these conceptions crumble when women incorporate themselves into the public sphere and into a paid job or into political participation [24,25]. Thus, a challenge emerges for women that want to have a family life as well as a job or any activity in the public sphere. These women are described as disruptive of domestic, community and social normativity [26,27,28].

Women that have a double working day dissolve traditional patterns of maternity. As such, they live in tension and unfortunately do not always find the support that they require in social structures to be able to develop in the professional field (for example, access to appropriate daycare or jobs that offer social security). Another issue is that women may never criticize, question or redefine traditional gender norms regarding a mother’s role, household load and child rearing [29,30]. This implies that women assume a traditional motherhood role, originating from a double working day in which, after a paid job, women continue to work without pay within their homes.

Because of the difficulties implicit in reuniting a job and a family, women who experience a double working day have developed different strategies and tools to tend to home and children while working. However, these strategies cannot guarantee that women perceive fulfillment and satisfaction because a sense of not having enough time to finish all of their tasks remains. Alcañiz [26] states that duplicity of functions entails angst, anxiety, restlessness, frustration, dissatisfaction, fatigue, resignation and even depression.

Our specific interest in the hospitality sector lies in the impact that a job in tourism generates. The ILO [31] recognizes that a fair job in the tourism sector is particularly challenging because of the employment conditions that characterize this sector: long hours (particularly in certain periods that are characterized by a surge in tourism), habitual synchronicity between work and breaks, employment seasonality, job informality, contract habits, non-habitual pay and a large percentage of low qualification positions occupied by women.

In line with this, different studies have previously exposed tourism as an element that inserts and perpetuates a power structure that favors gender inequality. In the context of Mexico, it has been found that the jobs of women in this sector constitute an extension of domestic labor—washing, cleaning, cooking, serving, etc.—but with economic remuneration [32,33,34,35,36,37].

Following these ideas, Vizcaino, Serrano, Cruz and Pastor [38] report that a strong tendency towards labor gender segregations prevails in tourism such that the different activities are differentially distributed as a function of gender roles attributed by society. This division follows social structures and dominant gender relationships. The authors also state that studies of the tourism sector should take the gender perspective into account so as to advance theoretical and conceptual debates on the dimensions of gender in this sector. It should also contribute to detecting problems that are specific to equal opportunities between men and women. It is important to highlight that the importance of this study is to expose the labor conditions of men and women specifically in the hospitality sector, thus contributing towards understanding the gender inequalities that we must presently confront.

There are various factors that can negatively or positively affect mental health or family functionality which are not related to working conditions; such factors can be age, context or economic situation. There is no one cause or explanation for such a complex psychosocial phenomenon. Understanding that various variables can be analyzed to explain mental health and wellbeing, the present study aims to focus on insecurity as an important factor that directly impacts mental health.

Considering this background, this study aims to compare working conditions, mental health, family functionality and job insecurity between men and women that are working in the hospitality industry. We will emphasize the inequalities that are found in this field of work. We will also analyze in what measure mental health, family functionality and job insecurity relate to each other and point out the predictive value of mental health variables.

## 2. Method

### 2.1. Participants

This research is a quantitative transversal study. Because the actual size of our population (the exact number of people working in the hospitality industry in Puerto Vallarta and Bahía de Banderas, Mexico) is unknown, our sample was selected through convenience sampling (also known as accidental sampling) in which the selection of the elements does not depend on probability but rather on what the study conditions allow for (e.g., access or availability) [39].

In non-probability sampling, the statistical unit is selected for convenience because of its availability. Thus, individual members of our population of study did not have the same probability of being selected. In these cases, the difference between the actual value of our population and the value of our sample is unknown and sampling error cannot be measured. However, convenience sampling can be more easily justified at the exploratory phase of a study as a means to generate hypotheses [40].

Our sample consisted of a total of 914 people working in hotels in Puerto Vallarta and in Bahía Banderas. Of these, 434 were women (this corresponds to 47.92%) and 476 were men (this corresponds to 52.08%). Participation was voluntary. Participants were informed that the data provided would be confidential. They were also informed that they had the liberty of abandoning the study at any phase.

### 2.2. Survey Tools

We used Likert scales and socio-demographic data forms:

In the socio-demographic form, we collected data on age, gender, nationality, residence, number of children, marital status, number of people living under the same room and family income. This form is of our own authorship but is based on the European Working Condition Survey (EWCS, 2010) created by Eurofound [41].

The Job Insecurity Scale (JIS-8) is a one to five Likert scale constructed by De Witte [42]. The original scale includes 11 items. However, when it was adapted to English, three items were eliminated. The version that we used is a Spanish validation of the scale created in the Universidad de Oviedo, Spain, by Llosa, Menéndez-Espina, Rodríguez-Suárez, Agulló-Tomás and Boada-Grau [43]. It has eight items that measure workers’ perspectives on job insecurity in two factors: cognitive and affective dimension. The confidence coefficients obtained were 0.72 and 0.84, respectively, as for 0.78 on the global scale.

The European Working Conditions Survey (2010) is a Likert scale survey with a response margin ranging from one to five, composed of 16 items, where elements relative to work realization and participation are evaluated. It is part of a larger survey created by Eurofound [41]. Only some aspects relative to working conditions relevant to the aims of this study were taken from this survey. In this study, we obtained a Cronbach alpha = 0.84.

The family APGAR questionnaire, with a Likert scale ranging from 1 to 5 and created by Smilkstien [44], was also used in its version that was validated by Bellón, Delgado, Luna del Castillo and Lardelli Claret [45]. This tool measures the perception of family function in five aspects: adaptability, partnership, growth, affection and capacity to resolve. The confidence coefficient obtained was 0.92.

The General Health Questionnaire (GHQ) created by Goldberg and Hillier [46] and validated by Retolaza, Mostajo, De la Rica, Díaz de Garramiola, Pérez de Loza, Aramberri and Markez [47], evaluates the general state, mental health or wellbeing in nonclinical populations. This version is composed of 28 items, with a Likert scale type response ranging from 1 to 4 that generates a global score, and 4 subscales: somatic symptoms, anxiety and insomnia, social dysfunction and severe depression. In this study, the confidence coefficients obtained were 0.83, 0.88, 0.79 and 0.89, respectively. The global score was 0.92.

## 3. Results

All of the analyses were conducted with statistical software SPSS version 22. We will first present descriptive data on the working conditions of men and women; then, we present Pearson correlations to determine the relationship between sub-factors of health with all of the studied variables. We also performed a *t* test to assess differences by sex. Finally, we performed a linear regression to examine the predictive values of variables related to global health. The following paragraphs detail the data that we collected together with the statistical analysis that we preformed.

### 3.1. Working Conditions Analysis

Here, we present our results. First, we illustrate the socio-demographic data and then we detail some of the working conditions of the survey participants.

As we mentioned earlier, 52.08% of our sample were men and 47.92% were women. Regarding family composition, 53.88% of women and 42.77% of men attested to having children under their care, implying that the majority of working women must reconcile family life and work. This is a challenge considering that the ILO [31] recognized that jobs in the hospitality industry are characterized by long working hours, non-traditional contracts, seasonality and situations that complicate the reconciliation of family life and work.

Single-parent families were more common in women. In total, 30.59% of women were single parents compared to 10.92% in men.

We asked about family composition in the homes of the surveyed people and found that 28.48% of men and 28% of women lived with their original families (father, mother and siblings), while 33.19% of men and 23.65% of women said they lived with a spouse and their children. These were two of the most predominant family compositions.

It is interesting to note that there were some notable differences between men and women. For example, the quantity of men that lived alone was double that of women (14.78% and 7.28%, respectively), the number of women that lived alone with their children was five times greater than that of men (16.94% and 3%, respectively), and the percentage of women that lived with a spouse or a friend was 1.5 times greater than that of men (4.24% and 2.27%, respectively).

**Figure 1:**
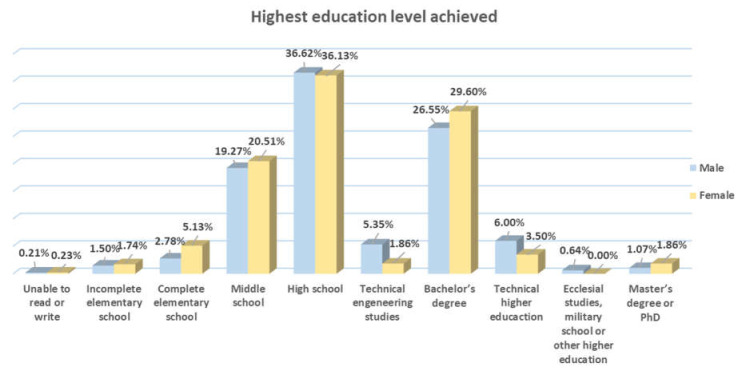
Highest education level achieved.

As for education level, we found that the predominant education level for both sexes was high school (36.72% of men and 36.13% for women), followed by bachelor’s degree level studies (26.55% of men and 29.60% of women). The most notable difference was in the area of engineering, where there was male supremacy because there were twice as many men as women (5.35% and 1.86%, respectively) in this area of study. We found the same pattern for technical higher education, in which 6% were men and only 3.5% were women. It is important to note that, in the area of elementary school, middle school and bachelor’s degrees, the percentage of women was greater than that of men (Figure 1).

**Figure 2:**
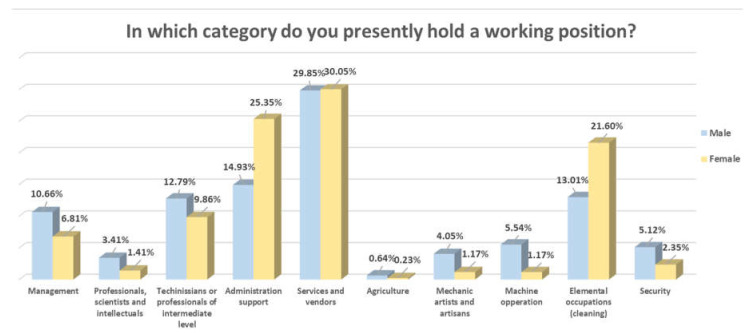
Category in which workers’ present positions were held.

Expanding on working conditions, we asked the surveyed participants about the category in which they presently worked. The predominant sector, both in men and women, was the service sector (waiters and waitresses, vendors, receptionists, bell boys, etc.). The category in which there were more differences was in administration support personnel (assistants or secretaries), in which women predominated. In the category of elemental occupations (housekeepers, laundry personnel, dishwashers, public area personnel, etc.), the percentage of women was also higher than that of men. The predominance of men in managerial positions, machine operations and security stood out (Figure 2).

**Figure 3:**
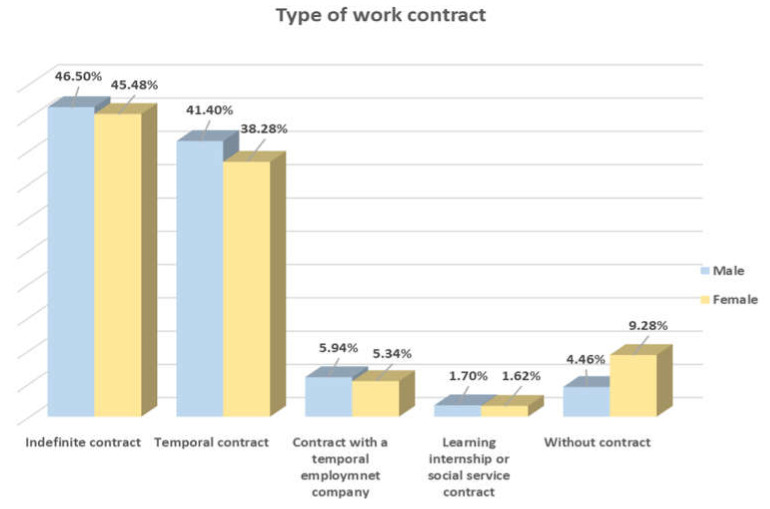
Contract type.

An important variable to analyze in working conditions is the type of contract between employees and company. On this subject, the high occurrence of temporary jobs stands out despite the fact that indefinite contracts are more common. Of people that were working without a contract, the percentage of women was greater than that of men (Figure 3).

Participants answered whether peers with the same position were men or women. In this aspect, 45.62% of women said that the majority of people with the same position as them were also women. In line with this, 51.60% of men said that people with the same position as them were men. Finally, around 30% coincided in the fact that people with the same position as them were more or less men and women in the same proportion, confirming gender segregation in the workplace.

Whether working hours adjusted well to social commitments and family outside of work, men and women coincided in the fact that their workday effectively adapted well or very well to commitments outside of their job; 41.75% of women and 43.31% of men said that their jobs adapted well to their social and family life. Contrary to this, 28.07% of women and 25.27% of men considered that their workday did not adapt well to commitments outside of their job. This is an interesting figure that gives us an idea of the understanding that they have of work and family conciliation.

In relation to monthly income, there was a difference between men and women such that men received on average 23.46% greater income than women. It is essential to consider that the education level between men and women was similar. Despite this, we still observed a disparity in income between the two sexes; the mean income of men was 12,913.18 Mexican pesos and the mean income of women was 10,458.75 Mexican pesos.

### 3.2. Correlations

Table 1 includes correlations between study variables, means, standard deviation and *t* tests. We found significant correlations between all variables. Somatic symptoms correlated significantly and positively with anxiety and insomnia, social dysfunction, serious depression and job insecurity. Family functionality also correlated significantly with these variables but the relationship was negative. We found significant differences between sexes in somatic symptoms (*t* = 4.59, *p* < 0.001), anxiety and insomnia (*t* = 4.21 *p* < 0.001) and family functionality (*t* = 2.22, *p* = 0.027). There were no significant differences between sexes in social dysfunction, serious depression and job insecurity. Women had higher means of somatic symptoms, anxiety and insomnia, while men showed a higher mean in family functionality.

### 3.3. Predictive Value of Global Health Variables

Lastly, regression results confirmed the predictive value of the mental health variables (Table 2). We confirmed that job insecurity (ß = 0.179; *p* < 0.001), family functionality (ß = −0.197; *p* < 0.001) and gender (ß = 0.129; *p* = 0.008) were variables which significantly explained mental health. Family functionality possessed the highest predictive value to health, with 18.7%, followed by job insecurity with 17.9% and worker gender with 12.9%.

### 3.4. Discussion and Conclusions

In this study, we aimed to comparatively analyze the labor conditions, mental health, family functionality and job insecurity of men and women working in the hospitality industry, with an emphasis on inequalities found in this field of labor.

Regarding labor conditions, our study found that the majority of women and men that were surveyed were in charge of young children and that traditional families were common. However, in many cases, women expressed that they were the head of their single-parent family. In other words, there is a large quantity of single mothers working in the hospitality industry of Puerto Vallarta and Bahía de Banderas. These women are in a greater state of vulnerability because they take full responsibility for raising children while at the same time maintaining a full-time job, as has been reported by many national and international organisms that confirm that women in the job market are confronted with inequalities such as lower income compared to men in a similar job, less opportunities to ascend to better positions at work and other forms of discrimination [17,18]. Future research directions could explore the strategies that these mothers take to make their jobs and their families compatible (e.g., the creation of support networks, access to daycare or putting older siblings in charge of younger siblings); it would also be interesting to analyze possible consequences that the pursuit of this compatibility has on physical and mental health.

Regarding education level, women most frequently achieved a bachelor’s degree. However, few educational levels showed significant differences between men and women; the most relevant differences were in engineering and technical degrees, which were dominated by men. These data reveal that there is not a great disparity in the education levels of the men and women that work in the hospitality industry and that, despite this, there are differences in the types of jobs that men or women occupy. We also found that the hospitality industry reproduces the gender structure by segregating women into basic cleaning positions that reproduce domestic labor. This has been previously documented by several authors who have found that this is common in the job market and that it is particularly common in the hospitality industry [32,33,34,35,36,37]. These findings moved us to question the motives behind employers assigning lower qualification positions mostly to women, regardless of their education level. This also led us to speculate that gender stereotypes play an important role in these types of decisions.

A characteristic that reveals that a job is precarious is the occurrence of temporal contracts or other unorthodox contractual forms, which may lead to job insecurity [1]. Thus, we highlight the fact that we found that 9.28% of women and 4.46% of men work without a contact. These findings should alert us to the insecurity that is experienced by people working in the hospitality industry and confirm that women are the ones that are most vulnerable to these conditions.

Together with these types of contracts, income also determines if a job is precarious or not. Our findings indicate that women receive on average 23.46% lower income than men; this shows that the job market is still plagued with gender inequalities which are detrimental to women.

Upon analyzing mental health variables, our results indicate that mental health relates negatively with family functionality and positively with job insecurity, which are systems that interact with and influence each other in public and private spaces. In other words, greater job insecurity results in lower mental health, and greater family functionality results in greater mental health. These results agree with the scientific literature that maintains that job insecurity has a direct impact on worker mental health [8,11,12,13] and is related to lower productivity.

It is important to deepen the significant findings which show that affective insecurity positively correlates with somatic symptoms and cognitive insecurity negatively correlates with family functionality. This can be explained in terms of a psychosocial model that establishes an interaction between the individual, the family, the job environment and social context. In other words, an individual experiences emotional insecurity, worrying about their working future, and somatizes the stress, resulting in symptoms of anxiety, depression and headaches, causing general health discomfort. In summary, the greater the emotional uncertainty, the greater the health problems; on the other hand, cognitive insecurity correlates negatively with family functionality, understanding that a person who perceives insecurity in their job experiences concern about the consequences of losing the job on the quality of family life, reducing their interaction, communication, harmonious coexistence and affecting their economy. Likewise, it is pertinent to clarify that cognitive insecurity does not correlate significantly with somatic symptoms, and emotional insecurity does not correlate significantly with family functionality, since they are two different factors that have different effects [43,48].

Women presented greater health problems with respect to men in areas such as somatic symptoms, insomnia and anxiety. The double working day saturates women and decreases women’s health. This produces fatigue, headaches, panic, trouble sleeping and feelings of being overwhelmed. These double hours affect women and manifest as symptoms of anxiety, angst, fatigue and dissatisfaction [26]. By incorporating themselves into a paid job, women defy beliefs, ideologies, values and conceptions [24,25] of gender stereotypes and use of power. However, society, community and domestic disruption [26,27,28] come at a cost to the psychological health of women, which is accompanied by different forms of labor [49,50], family and social discrimination.

Data confirmed that men have greater family functionality. They are satisfied with family life, which is understandable because they live in a patriarchal culture that favors their development in both public and private sectors and they do not endure a rupture in their assigned role. This makes men feel functional and normalized. Contrary to this, by working, women feel like they are not fulfilling their traditional role of being dedicated only to caregiving and caring for the household. As has been detailed by Díaz-Carrión (2013), Lara-Aldava and Vizcarra-Bordi (2008), Martínez (2003), Mendoza and Chapulín (2015), Soares, Castorena and Ruiz (2005) and Vargas (2010) [32,33,34,35,36,37], the tourism sector perpetuates the patriarchal structure of power and gender inequality in México. In the organizational functionality of the hospitality industry, labor segregation by gender prevails in the form of differentiating activities and positions mimicking social functions and roles [38].

At the same time, our results confirm that there is no difference in job insecurity between men and women. However, in the present study, there is a positive relationship between job insecurity and anxiety and insomnia, social dysfunction and serious depression. This means that by perceiving the cognitive and emotional danger of losing their jobs, workers in the hospitality industry suffer from general health issues. This result is in line with studies by Griep et al. [13], Landsbergis, Grzywacz and Lamontagne [11], Lee, Huang and Ashford [12] and Sverke [8], who state that there is a relationship between labor conditions and physical and mental health and social wellbeing.

As a matter of fact, our data show the importance of family functionality and the fact that job insecurity predicts general health. These findings support the importance of understanding the psycho-social phenomenon of workers from a systemic and social point of view and the link between individual, family and work, elements that should be taken into account in the process of training hospitality businesses and public policy making in the country. This should be done with the intention of improving labor conditions in the pursuit of gender equality, sustainable and inclusive growth [17] and fair job availability [1,2].

## 4. Recommendations

This study investigated and offered information on the interaction between several psychosocial variables such as family functionality, mental health and job insecurity. However, it is necessary to highlight that, because this is a transversal study, the results and conclusions obtained here would be stronger with a longitudinal study, which would allow us to establish causality in the relationships between the variables. In future studies, it would be important to integrate variables such as seniority, experience, type of contract and different socio-cultural contexts. Another limitation is the use of self-report for data collection, which can generate effects of social desirability and biases, although it is a valid and recommended measure in psychosocial studies [51,52]. To obtain greater accuracy, it is recommended for future research to use other sources of information, such as medical records, surveys or interviews of coworkers, partners and children, in order to contrast the results.

Despite these limitations, our findings allowed us to elucidate gender inequalities in the hospitality industry and their consequences for mental health and family functionality that could be used in writing public policies and fighting inequality in favor of improving workers’ quality of life both in males and females.

## Figures and Tables

**Table 1:** Pearson correlations, means, standard deviation and *t* tests between our variables. Asterisks denote significant results: * denotes a significant correlation with a *p* < 0.05 (two-tailed test), ** denotes a significant correlation with a *p* < 0.01 (two-tailed test), *** denotes a significant correlation with a *p* < 0.001 (two-tailed test).

		1	2	3	4	5	6	7
1. Somatic symptoms	Pearson Correlation	1						
	Sig. (two-tailed test)							
	*N*	897						
2. Insomnia and Anxiety	Pearson Correlation	0.661 **	1					
	Sig. (two-tailed test)	0.000						
	*N*	863	890					
3. Social dysfunction	Pearson Correlation	0.455 **	0.0493 **					
	Sig. (two-tailed test)	0.000	0.000					
	*N*	861	854	889				
4. Serious depression	Pearson Correlation	0.376 **	0.462 **	0.414 **	1			
	Sig. (two-tailed test)	0.000	0.000	0.000				
	*N*	853	847	846	882			
5. Family functionality	Pearson Correlation	−0.151 **	−0.211 **	−0.213 **	−0.268 **	1		
	Sig. (two-tailed test)	0.000	0.000	0.000	0.000			
	*N*	865	857	855	849	893		
6. Cognitive insecurity	Pearson Correlation	0.063	0.074 *	0.156 **	0.105 **	−0.184 **	1	
	Sig. (two-tailed test)	0.065	0.030	0.000	0.002	0.000		
	*N*	867	858	858	850	872	896	
7. Affection insecurity	Pearson Correlation	0.141 **	0.132 **	0.140 **	0.148 **	−0.056	−0.023	1
	Sig. (two-tailed test)	0.000	0.000	0.000	0.000	0.102	0.502	
	*N*	867	858	856	847	868	876	893
Male mean		4.85	4.69	5.31	1.80	20.09	9.40	10.52
Male standard deviation		3.57	4.20	3.13	3.31	4.96	3.32	4.22
Female mean		6.04	5.94	5.82	2.15	19.58	9.24	10.39
Female standard deviation		3.88	4.38	3.22	3.31	4.81	3.15	3.87
T		−4.59 ***	−4.21 ***	−2.14	−1.41	2.22 *	0.66	0.43

**Table 2:** Variables that predict global health.

Predictive Variables	Corrected *r*^2^	F	β	*p*
	0.083	12.72		
Job insecurity			0.179	0.000
Family functionality			−0.197	0.000
Gender			0.129	0.008

Note: Corrected multiple *r*^2^; F = Fisher’s F-Snedecor; β = Beta; *p* = α = 0.05.
